# When two biological species meet, will genetic exchange take place?

**DOI:** 10.1093/nsr/nwac292

**Published:** 2022-12-27

**Authors:** Hang Sun

**Affiliations:** Kunming Institute of Botany, Chinese Academy of Sciences, ChinaReviewer of NSR

Reproductive isolation (RI) is the core of biological species concept (BSC) [1]. In other words, there are many forms of RI among species that hinder genetic exchange to ensure genetic purity, so that species maintain their own biological characteristics. At the initial stage of speciation, the introgressions between species are usually a common phenomenon, which is more common in biodiversity hotspots [2,3]. However, after long-term isolation and divergence of species and formation of their own independent species according to their respective adaptive evolutionary paths, RI occurs among species and introgression is interrupted to form so-called biological species (BS). But if these species meet in sympatry, is it possible to break the RI and have genetic exchange? This is a long-term concern of evolutionary biologists.

Wang *et al.*’s [4] research confirms that such a situation exists. They selected two mangrove species, *Rhizophora mucronata* and *R. stylosa*, which have their own evolutionary history and distribution areas, they met in sympatry in northeast Australia during biogeographic expansion but still remain distinct species. They used *de novo* whole-genome assembly and re-sequencing of individuals across the range of two closely-related mangrove species that reveal the genomes to be well delineated in allopatry, however, it is found that their genomes harbor a small number of introgression blocks. This is very meaningful and important evidence that, despite different species’ secondary sympatry after long-term isolation and adaptation to different environments or areas, introgressions may still occur. This obviously challenges the BSC, and this study prompts us to rethink the definition of biological species.

Wang *et al.*’s [4] study showed there are indeed introgressions between the two BS. They suggest that BSC and the complete RI should be abandoned as a key criterion for BS delineation given the many recent genomic studies [3,5]. In fact, when defining the concept of biological species, it is often overlooked that there is a certain degree of RI between two species at the population level, that is, from 100% to 0% individual RI (from RI exists in all individuals to no RI exists) [6]. The evolution of species has always been a continuous process, the ideal BS should be the ultimate stage of species evolution, that is, 100% population level RI. Can we think that these two species *Rhizophora mucronata* and *R. stylosa*, are still on the way to biological species and have not yet formed biological species in the true sense?
